# Animal to human translation: a systematic scoping review of reported concordance rates

**DOI:** 10.1186/s12967-019-1976-2

**Published:** 2019-07-15

**Authors:** Cathalijn H. C. Leenaars, Carien Kouwenaar, Frans R. Stafleu, André Bleich, Merel Ritskes-Hoitinga, Rob B. M. De Vries, Franck L. B. Meijboom

**Affiliations:** 1 Department of Animals in Science and Society, Faculty of Veterinary Sciences, Utrecht University, Utrecht, The Netherlands; 2 Institute for Laboratory Animal Science, Hannover Medical School, Hannover, Germany; 3 SYRCLE, Department for Health Evidence (section HTA), Radboud Institute for Health Sciences, Radboud University Medical Center, Nijmegen, The Netherlands

**Keywords:** Translation, Prediction, Systematic review

## Abstract

**Background:**

Drug development is currently hampered by high attrition rates; many developed treatments fail during clinical testing. Part of the attrition may be due to low animal-to-human translational success rates; so-called “translational failure”. As far as we know, no systematic overview of published translational success rates exists.

**Systematic scoping review:**

The following research question was examined: “What is the observed range of the animal-to-human translational success (and failure) rates within the currently available empirical evidence?”. We searched PubMed and Embase on 16 October 2017. We included reviews and all other types of “umbrella”-studies of meta-data quantitatively comparing the translational results of studies including at least two species with one being human. We supplemented our database searches with additional strategies. All abstracts and full-text papers were screened by two independent reviewers. Our scoping review comprises 121 references, with various units of measurement: compound or intervention (k = 104), study/experiment (k = 10), and symptom or event (k = 7). Diagnostic statistics corresponded with binary and continuous definitions of successful translation. Binary definitions comprise percentages below twofold error, percentages accurately predicted, and predictive values. Quantitative definitions comprise correlation/regression (r^2^) and meta-analyses (percentage overlap of 95% confidence intervals). Translational success rates ranged from 0 to 100%.

**Conclusion:**

The wide range of translational success rates observed in our study might indicate that translational success is unpredictable; i.e. it might be unclear upfront if the results of primary animal studies will contribute to translational knowledge. However, the risk of bias of the included studies was high, and much of the included evidence is old, while newer models have become available. Therefore, the reliability of the cumulative evidence from current papers on this topic is insufficient. Further in-depth “umbrella”-studies of translational success rates are still warranted. These are needed to evaluate the probabilistic evidence for predictivity of animal studies for the human situation more reliably, and to determine which factors affect this process.

## Background

The general aim of biomedical research is to develop possible cures for diseases. Current drug development is handicapped by high attrition rates; many molecules that were promising during preclinical development fail during subsequent clinical testing [1]. At the moment, return on investment in pharma is lower than ever [2]. Part of the attrition may be due to low animal-to-human translational success rates; the so-called “translational failure” [3].

There are two fundamental perspectives potentially explaining translational failures. The first main perspective is that the concept of animal-to-human predictability is fundamentally mistaken [4]. This perspective is based on the observation that the hypothesis that animals are predictable for humans has never been scientifically tested [5, 6], and that there are important differences between species in e.g. physiology, genetics, epigenetics and molecular biology [4, 7]. Animal studies have historically been implemented in drug approval procedures, which may have been based on scientifically outdated principles [8]. Besides, animals and humans are complex systems, that are more than the sum of their parts, and therefore always unpredictable [9, 10]. From this perspective, animal experiments that are performed to inform human health are not ethically acceptable.

The second main perspective is that biomedical and pharmaceutical research advanced over the last decades because animal experiments are in general able to predict the situation in humans [11]. In this perspective, recent translational failure could be explained by suboptimal experimental design [12, 13], and lack of reproducibility in general [1]. Many of the factors involved in suboptimal design of animal studies and the resulting bias, have been reviewed before, and are increasingly taken into account by the scientific community [14–19].

Both perspectives are currently promoted by different groups of scientists. Neither group routinely refers to the total body of available evidence on animal-to-human predictability. This predictability, i.e. the translational success rates, can be determined quantitatively in various manners. For example, researchers can sample clinical trials from a registry, retrieve the supporting preclinical data and analyse to what extent the data correspond. Alternatively, they can sample preclinical studies with relevance to humans, and analyse subsequent clinical studies. Moreover, researchers can analyse the effects of a set of interventions (e.g. drugs) on specific outcomes (e.g. biochemistry, physiology and adverse events) in multiple species.

Several methods have been used to analyse translational success, and many authors have addressed the problem of attrition in translational research, e.g. [3, 16, 20]. Most of the papers published on the topic provide expert opinions, narrative reviews or primary studies showing mechanistic similarities between species. As far as we know, no proper overview of the actual data is currently available. While the debate will not be decided by these data alone, an overview of the observed predictive values in different data sets will aid the ethical discourse on the acceptability of those animal experiments intended to inform decisions for human exposure.

In medicine, systematic reviews (SRs) have long been considered to provide the highest level of research evidence, as they combine all available data [21]. In animal research, SRs are increasingly used to collate all available evidence on a subject using transparent and reproducible methodology. We set out to summarise all available published evidence on animal to human translational success. Due to the lack of specific and sensitive indexing of this type of studies, performing a full comprehensive search to retrieve all available studies was not viable. We thus performed a systematic scoping review. Scoping reviews aim to estimate the size and quality of literature on a topic [22]. In the present systematic scoping review, we fully analysed the included papers, to summarise quantitative data from studies that assessed animal-to-human translational success rates.

The main question was “What is the observed range of the animal-to-human translational success (and failure) rates within the currently available empirical evidence?” In contrast to a full systematic review, our review did not follow the PICO format for outcome measures as we included all relevant outcomes, and it did not comprise a full comprehensive systematic search. The search was supplemented by alternative strategies, as detailed in “Methods” section.

Besides studies explicitly addressing translational success rates, we included meta-analyses comprising both human and animal data, and studies analysing the correlation of similar outcomes between animals and humans, as they provide quantitative information on translation for individual interventions. As far as we are aware, we are the first to provide a systematic scoping review of all types of published findings (i.e. literature reviews and other types of “umbrella”-studies) on quantitative analyses of animal-to-human translational success.

## Methods

The protocol for this systematic scoping review was posted online on the SYRCLE website (http://www.SYRCLE.nl) on 27 December 2017 [23], after performing the Pubmed and Embase searches, but before the start of paper selection.

### Research question

The main research question for this systematic scoping review was: “What is the observed range of the animal-to-human translational success (and failure) rates within the currently available empirical evidence?”. We originally defined translational success as “replication in a randomized trial in humans of statistically significant positive, negative or neutral results for the primary study outcome in animal experiments”, and consequently, translational failure as not replicating the results of animal experiments in a randomized trial for the primary study outcome. We did not expect to find clinical trial publications after animal experiments with negative or neutral efficacy results, nor did we expect to find many after positive toxicology results.

We intended to preferentially address studies on phase I–II trials to focus on early clinical trials over market access, as successful early trials do not always result in clinically available medication for reasons beyond animal-to-human predictability. In practice, only few of the included references detailed the types of trials and experiments, or the primary study outcomes. During the screening of the retrieved references, these two elements thus had to be disregarded. Our working definition of translation therefore became “the quantitative degree of correspondence between the results from a trial in humans with results in animal experiments”. This was communicated between the screeners. We did not post an amendment to the protocol because we did not expect this broader definition to increase bias in the selection of studies and thereby the results.

### Search

Our search consisted of 3 elements: animal models, human trials, and translation. We first tested several traditional comprehensive search strategies based on both medical subject headings (MeSH) and on words in the title, abstract and keywords in PubMed. Regardless of the exact combination of search terms used, the number of references retrieved became too high to manage within the timeframe of this project. We thus went for a less conventional scoping strategy, searching for MeSH-terms and title words only. As we expected to miss relevant literature this way, we introduced additional search strategies (detailed below).

Our final search for Pubmed consisted of MeSH-terms and title words (including several synonyms) for animal models, human clinical trials and translation, combined with “AND”. We built an equivalent search for Embase (replacing MeSH terms with the corresponding Emtree terms), also including key words. We filtered for (systematic and other) reviews, letters and editorials. The full search strategy can be found in our protocol [23]. We performed our systematic scoping search in Pubmed and Embase on 16 October 2017.

### Additional search strategies

Besides formal literature searches, we retrieved relevant references via two more routes. The first was screening of the reference lists of all included references and relevant reviews. This is a standard approach in systematic reviews. The second alternative route was contacting experts in the field for additional references. Experts were (1) the authors; (2) their (direct and indirect) colleagues known to be interested in translational success, and (3) the first and last authors of all papers included from the search. Experts were contacted via email; a single reminder was sent after 1–2 weeks if they did not respond.

### Selection of papers

We included studies and reviews quantitatively comparing the results of studies including at least 2 species with one being human. We thus excluded studies and reviews comparing 2 non-human species or comparing outcomes between human clinical trials. There were no restrictions for language or publication date. All titles and abstracts retrieved from the search were independently screened by two reviewers. Full-text screening of the included papers was again performed by two independent reviewers. Discrepancies were resolved by discussion between reviewers.

During the selection process, we came across several correlational studies of pharmacokinetic–pharmacodynamic (PKPD) parameters after the administration of various molecules. While these papers do not describe translational success rates according to our original binary definition (replication of positive, negative or neutral results), they do provide continuous quantitative information on animal-to-human translation. As this is in line with our intended goal, we did include these papers. The same argumentation led to the inclusion of meta-analyses in which both human and animal studies were compared as subgroups within a single meta-analysis.

Comparisons of outcome measures without an intervention were excluded (e.g. [24–26]), as well as papers describing the effects of experimental design parameters on outcomes in several species (e.g. [27]. Ex vivo and in vitro animal studies were also excluded (e.g. [28, 29], as well as animal studies combining the animal with other (mostly in vitro) data to improve predictive accuracy [30]. We only included studies that provided quantitative information on translational success (or failure); i.e. we excluded papers comparing a single human with a single animal study. The unit of analysis could vary, but included studies had to compare a specific set of compounds/interventions, studies/experiments, or symptoms/events between species. The important work of O’Collins et al. [31] was excluded from our analyses as their efficacy comparison between species is not based on the same set of drugs.

Besides, several important reviews focusing on translation from the animal study perspective only were excluded (e.g. [32–36]), as well as studies analysing how often animal studies were cited [37]. Further excluded were important papers on attrition rates and translation with a wider scope than animal-to-human translation (e.g. [3, 38–46]), papers presenting relevant graphs without informing us on summary values (e.g. [5], and quantitative studies on related phenomena such as market withdrawal of drugs [47] and animal harm–human benefit analyses [48].

### Selection of data

When a single paper described multiple studies on different datasets, those compliant with our inclusion criteria were included into the analyses separately. E.g. [49] described 3 studies of which 2 are included in this review; the 3rd study, on intestinal expression levels of transporters and metabolic enzymes in rats and humans, did not comprise an intervention and was thus excluded.

If species were analysed separately, we included the separate data. If multiple analyses with the same outcome measure were based on the same data, we included the one with the largest sample size (which was deemed the most reliable), or, in a minority of studies with equal sample sizes, the most predictive one (i.e. the highest translational success rate). Including the most predictive results may have biased our results somewhat towards inflated translational success rates. For PKPD studies reporting ≥ 3 parameters, we preferentially selected the volume of distribution, the clearance and the half time, as these were most frequently reported.

For papers describing several meta-analyses based on the same studies, the primary outcomes were selected. If no primary outcome was described, again, the largest analyses were preferentially selected. If multiple binary analyses were based on the same data, we preferentially included the accuracy (see Table 1). If negative and positive predictive value (see Table 1) were both provided without the crude data (from which we could have calculated the accuracy), we included both values.

**Table 1:** Diagnostic statistics with binary definitions of translational success. Adapted from e.g. [8]

	Human test	
	Positive (HP)	Negative (HN)
Animal test		
Positive (AP)	TP	FP
Negative (AN)	FN	TN
Sensitivity = TP/HP		
Specificity = TN/HN		
Positive predictive value (PPV) = TP/AP		
Negative predictive value (NPV) = TN/AN		
Accuracy = (TP + TN)/(TP + FP + FN + TN)		

*AN* animal negative, *AP* animal positive, *FN* false negative, *FP* false positive, *HN* human negative, *HP* human positive, *TN* true negative, *TP* true positive

### Analyses of translational success rates

As we observed a large range of reported translational success rates, we exploratively analysed the data further. However, different papers used different strategies for quantifying translation. It is important to realise that different definitions for translational success result in different diagnostic statistics, which may result in different values for the same data.

Different diagnostic statistics lead to different predictive values, even when based on the same data, which we included as described in the preceding section. The differences are clear for e.g. the percentage < twofold error versus the correlation [50], and for the sensitivity, specificity, positive predictive value and negative predictive value [51]. The data are not always so discrepant. For example, the percentage of overall correct predictions reported by Litchfield is 74% when both rat and dog are considered [52]. We can also use his data to calculate specificity (72%), sensitivity (76%), positive predictive value (68%), and negative predictive value (79%).

The diagnostic statistics can be clustered into two main categories: continuous (degree of comparability in effect size; e.g. correlation or  % overlap in confidence interval) and binary (translation yes/no; e.g. percentage accurately predicted or percentage below twofold error). For a direct comparison of continuous outcomes, analyses of correlation and regression were common. For yes/no type decisions, several binary classification measures were used, as described in Table 1.

Besides various analyses resulting in different values, different types of values for translational success have different meanings. For example, when analysing percentages of binary success/failure rates, 50% is equivalent to tossing a coin, while 50% overlap in a confidence interval (meta-analyses), or 50% explained variance (correlation and regression) can be considered to result in meaningful data.

If the authors of a paper did not provide a summary measure for translation, we calculated one where we could. For example, for a study on the predictive validity of pain models [53] we calculated the correlation coefficient for the maximum plasma concentration at the minimum effective dose in rats and the maintenance dose in humans using Excel. When different sources were provided describing different values for a single data point (e.g. different values for a single drug in a correlational analysis), we used the median.

All included studies were tabulated. In our tabulations of study outcomes, we aimed to summarise the data and reflect the original authors’ view. To summarise our findings quantitatively, we expressed all values for translation as percentages. The conversions are described in Table 2. For correlations and regression analyses, we selected r^2^ over r, as this value reflects the percentage of explained variation. When both correlation and % < twofold error were presented, we selected r^2^ for inclusion in the analyses as these values better reflect the actual data. Similarly, when binary classifications were provided, we preferentially selected accuracy, or, when accuracy was not given, the positive and negative predictive values. For meta-analyses, we determined the degree of overlap of the animal 95% confidence interval (CI) with the human 95% CI. There was only one study where the 95% CIs did not overlap, and in that study, the direction of the effect was opposite in animals compared to humans, included in the analyses as 0% translational success.

**Table 2:** Measures used to reflect translational success rate

Type of analysis	Type of definition for translation	Used value for % translational success
Correlation	Continuous	Squared correlation coefficient (r^2^) expressed as percentage
Regression	Continuous	Squared correlation coefficient (r^2^) expressed as percentage
Fold error	Binary	Percentage below twofold error
Meta-analysis	Continuous	Percentage overlap of 95% CIs
Binary analyses	Binary	% accurate or PPV or NPV

*95% CI* 95% confidence interval, *PPV* positive predictive value, *NPV* negative predictive value

To visualise the variation in reported translational success rates, we plotted all values from all included studies in a histogram. We then created boxplots with the individual data points in overlay. Plots were created in R version 3.5.0—“Joy in Playing” [54], using the GGPlot2 package [55].

### Risk of bias and reporting quality

According to the protocol, we analysed risk of bias and reporting quality of the included references for the following items: power calculation for the translational comparison, sampling method of the studies included in the analysis, type of data analysis, blinding in the sampling procedure, blinding of the data analyst, control for publication bias (i.e. did the authors analyse the effect of potential underreporting of small neutral studies in their estimate of the translational success outcome), risk of bias analysis performed for each of the included studies, and overall risk of bias estimate. For each of these items, we separately analysed if they were reported, and if there were resulting risks of bias (yes/no/unclear). Of note, power analyses are not common in literature reviews, as systematic reviews aim to include all available evidence, and with complete sampling, power calculations become irrelevant.

Besides, as the included papers described some type of review of the literature, we analysed their compliance with the PRISMA (Preferred Reporting Items for Systematic reviews and Meta-Analyses) guidelines for the following items (being aware that these guidelines do not necessarily apply to other review types): registration of a protocol, explicit description of eligibility criteria, the number of screeners determining which papers to include, the number of scientists performing the data-extraction, whether an analysis of risk of bias was performed on the included studies individually and overall, if the analyses had been prespecified, if the limitations of the review were discussed, and if the funding was described. At the time we posted our protocol, the more relevant PRISMA extension for scoping reviews [56] had not yet been published.

For funding, we further estimated whether there was a potential risk of funding bias, which could go either way. We scored a high risk of funding bias if the funder was indicated as or if any of the authors worked for a non-governmental (animal rights) organisation, a pharmaceutical company, or a governmental regulating agency (e.g. EMA, FDA).

Only those relevant PRISMA items not otherwise analysed were extracted. E.g. item 13 and 14 (summary measures and synthesis of results) overlap with our extracted data on type of analysis, and e.g. items 1 and 2 (title and summary) were not deemed relevant for the overview provided in this paper. The item publication bias can be considered irrelevant for certain types of review, for example when internal databases are used. Where included references were not based on publications, we reinterpreted this item for the type of data included, e.g. the risk of studies not ending up in the internal database from which a dataset was extracted.

## Results

### Search and selection

**Fig. 1:**
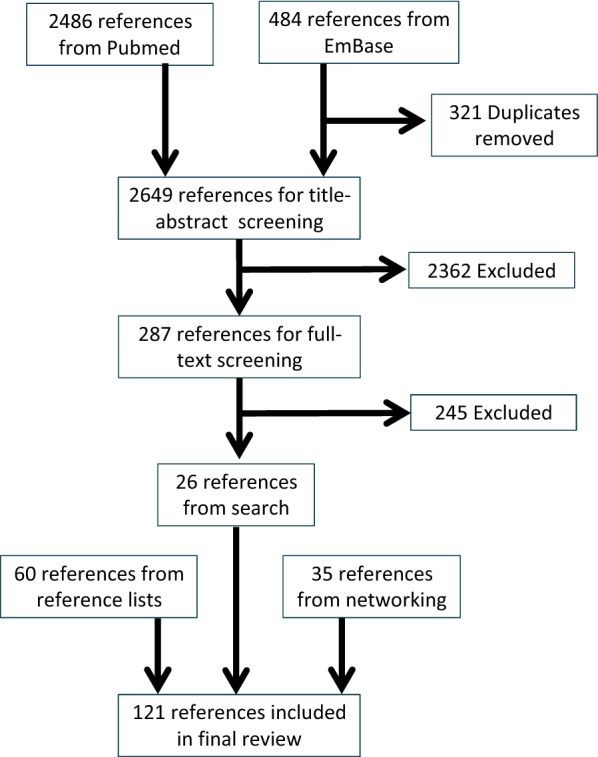
Flow scheme of papers

Our search in PubMed retrieved 2486 references, that in EmBase retrieved 484. After duplicate removal, 2649 references remained, after title-abstract screening, 287. After full-text screening, 26 references were included in this review. Screening the reference lists resulted in 60 additional references. Contacting the first and last authors of the 26 references included from the search combined with contacting people in our network resulted in 35 additional references. The flow of papers is shown in Fig. 1.

### Characteristics of the included papers

Of the 121 included references, 119 were in English, one was in German, and one was in French. The unit of measurement was compound or other type of intervention for 104 references, study/experiment for 10, and symptom or event for 7. The number of included interventions, studies or symptoms per reference ranged from 5 to 1256 (also see Fig. 8). Specific animal models were described in 35 references, and comprised e.g. xenografts, bile duct cannulated animals, chimeric mice, or a combination of various models.

Reporting information was limited; less than 15 references reported sex, age or disease status of the animals or humans included in the analyses and the type of studies or trials. Information on dose was reported in 24 references, information on route of administration in 40 (mainly multiple and intravenous, also oral, intraperitoneal and topical). These data were not further analysed.

### Studies addressing translational success rates

#### Studies addressing general medical sciences and efficacy

Of the 121 included references, 16 addressed efficacy or translation in general. The results from these references are summarised in Table 3. Several of these references were familiar to the authors before starting this work and provided the background for our protocol [57–59]. Lindl et al. followed the results from 51 animal ethics requests, and found very little translation to the clinical situation [58], with their analysis restricted to a 10-year time window. This may be rather short for analysing translational success, as the development of new treatments is a lengthy process, (see e.g. [39]) and development times seem to increase over time [2]. Hackam et al. followed highly cited animal studies, and found that about one-third translated to randomised clinical trials [57]. Perel et al. compared the effects of 6 interventions between animals and humans with systematic literature reviews, with half of them concordant [59]. We hoped to retrieve a number of comparable references, but only found one; Contopoulos-Ioannidis et al. analysed 101 articles that described novel therapeutic or preventive promises based on animal data [60]. 16 of these novel interventions were tested in clinical trials, of which 12 had a positive result in the trial.

**Table 3:** References on translational success in general and in efficacy studies

Study ID	Field of research	Summary of findings
Briassoulis_2014	Sepsis	Animal studies show clear protective effects of HSP72 in sepsis, human studies are inconclusive
Brossi_2015	Orthopedia	Equine studies on the efficacy of platelet rich plasma (k = 63) mostly show positive results, human studies (k = 60) have variable outcomes. Beneficial results are more frequent in studies with a high risk of bias
Contopoulos-Ioannidis_2003	Diverse	Out of 64 publications of animal studies in highly cited basic science journals, 16 interventions were tested in a published clinical trial, 12 of which had positive results
Corpet_2005	Oncology	Relative Risks after treatment were discordant for 2 out of 11 compounds between rats and humans, and mice and humans
Faggion_2009A	Dentistry	pocket depth reduction and attachment level gain were similar for animals and humans
Hackam_2006	Diverse	Successful translation is not predicted by study methodology, but it is predicted by the presence of dose–response gradients in animals
Johnson_2001	Oncology	Xenograft models that were available at this stage could not reliably predict the clinical response
Lindl_2005	Diverse	The publications resulting from 51 animal ethics approvals were followed. 16 projects were relevant to humans and resulted in 63 publications that were cited 1183 times. 97 citations were clinically oriented, of which only 4 evidenced an animal-human correlation. The hypotheses verified in animals failed in every respect in humans
Perel_2006	Diverse	For 6 interventions, animal and clinical studies were concordant for 3 and discordant for the other 3
Steinberg_1987	Pancreatitis	With the same 5 interventions, 81% of animal studies had a positive outcome, and only 7.7% of the human studies.
Sultan_2017	Cardiology	Most of the human data did not show any effect of cannabidiol, while the animal studies did
Valles_2018	Dentistry	Results from animal and human studies are concordant
Voskoglou-Nomikos_2003	Cancer	None of the primary analyses showed a significant correlation
Whiteside_2008	Pain	For effective pain treatments, the correlation between human and rat effective doses is good
Yardley_2016	Alcohol abuse	Out of 49 animal studies (on 8 drugs), 45 showed positive results. Out of 76 human studies, 56 showed positive results.
Yen_2014	Dentistry	Animal models and human results showed similar bone filling ratios

*K* the number of included studies

Three references compared the number of positive-outcome studies between animals and humans for similar interventions [61–63]. Four other included references comprised meta-analyses showing both human and animal data [64–67].

The included analyses comprise correlational analyses (R^2^), the Chi-square test, relative risk, accuracy and meta-analyses.

#### Studies analysing adverse events and toxicology

Of the 121 included references, 28 addressed translation of safety studies. Adverse events were analysed in 17 of these, carcinogenicity in 6. The other 5 references described translation for drug-induced liver injury, QT prolongation, skin sensitization, teratogenicity and toxic dose. The included references comprise analyses of concordance, likelihood ratios, positive and negative predictive values, sensitivity, Chi-square and correlation. The results from these references are summarised in Table 4.

**Table 4:** References on translational success in studies of adverse events and toxicology

Study ID	Field of research	Summary of findings
Alden_2011	Carcinogenicity	Out of 287 registered drugs that were tested in rats and mice for carcinogenicity, results were concordant with humans for 146
Allen_1988	Carcinogenicity	Correlation of carcinogenic dose between animals and humans ranged from 0.49 to 0.90 depending on the analysis
Bailey_2013	Safety	All likelihood ratios (LRs) are larger than 1, indicating predictive value of the experiments in dogs. Inverse negative LRs (iNLRs) are very small, indicating relatively limited predictive value of negative results in dogs for humans. Positive LRs (PLRs) for dogs are large; if toxicity is observed in dogs, it is likely to occur also in humans. There is no correlation between positive predictive values (PVVs) and PLRs
Bailey_2014	Safety	All LRs are larger than 1, indicating predictive value of the experiments in rats, mice and rabbits. iNLRs are very small, indicating relatively limited predictive value of negative results in these species for humans. PLRs for these species are large; if toxicity is observed in rats, mice or rabbits, it is likely to occur also in humans. Both PLR and iNLR depend on sample size
Bailey_2015	Safety	All LRs are larger than 1, indicating predictive value of the animal experiments. iNLRs are very small, indicating relatively limited predictive value of negative results in animals for humans. PLR for non-human primates (NHPs) is large; if toxicity is observed in NHP, it is likely to occur also in humans
Brown_1983	Teratogenicity	Correct positives: 30–97%; correct negatives: 35–80%; animal to human lowest effective dose ratio: 1.8–50
Claude_2007	Adverse events	70% of human adverse events was predicted by animal models. Predictivity is higher for non-rodents than rodents. Predictivity was highest for haematological and cardiovascular, and lowest for cutaneous and ophthalmological adverse events
Crouch_1979	Carcinogenicity	Data for carcinogenic potency correlated
Davis_1998	QT prolongation	Out of 9 noncardiac drugs that show QT prolongation in humans, literature on dog cardiac effects was found for 7; 6 showing QT prolongation, 1 showing increased mortality
Ennever_2003	Carcinogenicity	Sensitivity appears to be high, but the lifetime rodent bioassay lacks accuracy. Sensitivity decreases if only results that are positive in both rats and mice are considered positive. The LRB produces many false positives and false negatives
Fletcher_1978	Adverse events	Correlations between animal toxicity and human adverse events are considerably more frequent than discrepancies. Gastro-intestinal adverse events show the best correlation
Fourches_2010A	Drug-induced liver injury	The concordance of liver effects between rodents and humans (44%) and between non-rodent species and humans (40%) was low
Freireich_1966	Toxic dose	Results in preclinical tests correlate remarkably well with results in man
Goodman_1991	Carcinogenicity	For 18 out of 20 examined chemicals with sufficient evidence, human and rodent evidence are consistent
Hoffmann_2018	Skin sensitization	Overall accuracy in skin sensitization prediction from animal to human was 74%, which decreased to 45% when considering five categories of potency
Igarashi_1995	Adverse events	Out of 31 pharmacological items tested after systemic administration, 17 showed a significant association with any clinical adverse reaction
Litchfield_1961A	Adverse events	18 out of the 53 physical signs observed in man were predicted correctly in rats; 29 out of the 53 in dogs
Litchfield_1962	Adverse events	Out of the 86 physical signs analysed in animals, 64 accurately reflected occurrence or absence in man
Monticello_2017	Adverse events	Excluding subjective adverse events, for rodents, PVV ranged from 0 to 54% and NPV ranged from 69 to 96%; for dogs, PVV ranged from 0 to 52% and NPV ranged from 76 to 96%; and for monkeys, PVV ranged from 0 to 91% and NPV ranged from 70 to 100%
Olson_2000A	Adverse events	In any species tested, 71% of human adverse events was predicted. Predictivity is higher for non-rodents than rodents. Predictivity was highest for haematological, cardiovascular and gastrointestinal toxicities, and lowest for cutaneous toxicities
Schein_1970	Adverse events	For the prediction of certain adverse event in humans, administration of highly toxic dose levels to animals is needed
Schein_1973a	Adverse events	For most organ systems, combining dog and monkey data reduces false negatives for prediction of human adverse events for anticancer drugs
Schein_1973b	Adverse events	Correct predictions of anticancer drug-induced adverse events are accompanied by a high percentage of false positives
Schein_1975	Adverse events	Results from 13 additional drugs generally overlap with the preceding analysis
Tamaki_2013	Adverse events	37% of adverse drug reactions in humans were predicted from animal studies
VanMeer_2012	Severe adverse reactions	Performed animal studies are not sensitive enough to predict post-marketing serious adverse reactions
Weaver_2003	Adverse events	No significant associations were observed between human and guinea pig data
Wilbourn_1986	Carcinogenicity	Sensitivity for the predictivity of animals for human carcinogenicity is high (84%), and there is good consistency between animals and humans in target organs

Of note, the 4 references (comprising 6 studies) with sample sizes over 200 all fall within this category [68–71]. Tamaki et al. studied 1256 adverse drug reactions after administration of 142 drugs that were approved in Japan from 2001 to 2010 [71]. 48% of the adverse drug reactions could be predicted from the animal data. Fourches et al. mined the literature to create a data set of 951 compounds with effects in the liver in different species [69]. The concordance of liver effects between animals and humans was relatively low. Olson et al. described 221 human toxicity events after administration of 150 (coded) compounds, as reported by 12 pharmaceutical companies [70]. The concordance rates between animal and human toxicity were 71% when all species were considered, with nonrodents alone predictive for 63% and rodents for 43% of the events. Alden et al. reviewed drug labels from the physicians’ desk reference, which they searched for any mention of terms related to carcinogenesis [68]. This resulted in 533 active pharmaceutical ingredients that were further analysed. Of these, 287 had been tested in rodents, in which 161 tested positive for carcinogenicity. The authors presented the sensitivity (73%), positive predictive value (20%, refer to Table 1 for an explanation of predictive values; true positives are in this case ingredients that show carcinogenicity in animals and humans), negative predictive value (90%) and crude data.

#### Studies addressing pharmacokinetics

Of the 121 included references, 77 addressed translation of various pharmacokinetic (PK) parameters, mainly clearance, bioavailability, volume of distribution and concentration–time profiles. The results from these references are summarised in Table 5. Besides animal–human correlations for PK values from several drugs, these studies often analyse the fold-error of the predicted compared to the observed value, and the percentage of compounds with a predicted value within twofold of the observed value.

**Table 5:** References on translational success in pharmacokinetics

Study ID	Field of research	Summary of findings
Akabane_2010A	Absolute bioavailability	Bioavailability in cynomolgus monkeys is unsuitable for predicting PK in humans
Bachmann_1989	Clearance	Predicted values are in the same order of magnitude as actual values
Bachmann_1996A	Volume of distribution	Human volume of distribution and half-life values can be predicted from those in rats
Boxenbaum_1982A	Clearance	It is not possible to reasonably predict human pharmacokinetic parameters from knowledge of these parameters in dogs
Caldwell_2004A	Clearance	There is a reasonable correlation between human and rat clearance and half-life; and a good correlation for volume of distribution, but only 52–65% of drugs show < twofold error. Go/no go decisions based on only rat data should be avoided
Campbell_1994A	Clearance	Predictive accuracy for clearance from rat, dog and monkey is acceptable. The dog is a poorer predictor of clearance than the rat
Cao_2006A	Oral bioavailability	Oral bioavailability does not correlate between rats and humans; R^2^ = 0.29 while intestinal permeability correlates better; R^2^ = 0.70
Cheng_2008	Oral absorption	Human intestinal absorption cannot be precisely predicted by a single screening assay
Chiou_1998A	Oral bioavailability	Oral bioavailability correlates between rats and humans, and to some extent between dogs and humans
Chiou_2000a	Oral absorption	Similar gastrointestinal absorption may be obtained when doses in humans (/kg body weight) are 5–7 times lower than in rats
Chiou_2000bA	Oral absorption	R^2^ = 0.51–0.90 for oral absorption between dogs and humans; plasma level peak times seem to be shorter for dogs. R^2^ = 0.95 for oral absorption between rats and humans.
Chiou_2002A	Oral absorption	Oral absorption correlates well between monkeys and humans: R^2^ = 0.97; bioavailability correlates to some extent between monkeys and humans: R^2^ = 0.50; clearance correlates between monkeys and humans: R^2^ = 0.82; time to peak concentration was generally similar in humans and monkeys
DeBuck_2007A	Volume of distribution	Predictions of plasma concentrations after oral dosing are reasonable. Prediction of volume of distribution improves when accounting for interspecies differences in plasma protein binding. 18 out of 19 drugs had a predicted half-life within twofold of the actual observed half-life
Dong_2011A	Volume of distribution	For Monoclonal antibodies with non-linear kinetics, prediction is poor, with up to 6.3-fold differences
Evans_2006	Clearance, distribution volume and residence time	Percentages of correct predictions for clearance, distribution volume and residence time for rat, dog and monkey varied from 29 to 91%, and the average margin of error from 44 to 159%. The authors note that the outcomes are different from similar analyses of other compound datasets
Fagerholm_1996	Jejunal permeability	For passively absorbed compounds (n = 8), the correlation is high; R^2^ = 1.0. For passively absorbed compounds, rat permeability estimates can be used to predict human oral absorption
Fagerholm_2007a	Fraction excreted unchanged	Out of 25 compounds, 11 had a fraction of 0 excreted unchanged in both humans and rats. For 9 out of 14 compounds with renal excretion in rats and humans the major route of elimination differed between species. Findings for monkey–human comparisons were roughly comparable
Fagerholm_2007b	Unbound fraction in plasma	The fraction unbound in plasma correlates between rats and humans; R^2^ = 0.67. Different prediction methods show different accuracies
Goteti_2010A	Clearance	Two-species scaling can be useful, but the prediction of clearance from ≥ 3 species is more accurate
Grime_2013A	Clearance	For 19 out of 22 drugs, rat unbound biliary clearance exceeded human clearance by factors ranging from 9- to 2500-fold. Human–dog differences in biliary clearance were less dramatic than human-rat differences
He_1998A	Oral bioavailability	In human and rat there is generally a good correlation for oral bioavailability, in human and dog there is no apparent correlation.
Hosea_2009A	Clearance	Single species scaling is as accurate or more accurate than multiple-species allometry
Ito_2005A	Intrinsic clearance	Human clearance is better predicted by modelling based on in vitro microsomal data than on animal data
Jolivette_2005A	Clearance, volume of distribution	Molecular properties may be used to improve extrapolation from animal to human clearance
Jones_2012A	Clearance, mean residence time	Prediction was within twofold for 5 out of 7 compounds
Jones_2016C	Intestinal availability	There is little evidence that one animal species is sufficiently predictive of human first-pass metabolism to be used as a stand-alone model
Kalvass_2007A	In vivo potency (EC50), clearance	In vivo mouse brain half-lives are almost identical to human half-lives. In vivo preclinical to clinical extrapolations are superior to extrapolations from in vitro tests
Lave_1999	Clearance	Human clearance is most accurately predicted from a combination of in vivo animal and in vitro animal and human data
Lave_2002	Clearance	Predictions based only on in vitro data are at least as accurate as those based on multiple species data
Lennernas_2007	Jejunal permeability	A rat model can be used to predict oral drug absorption, but not drug metabolism or oral bioavailability
Ling_2009	Clearance	Human clearance might be accurately predicted from monkey data
Mahmood_1996a	Clearance	Human clearance can be estimated from animal data, but caution and scientific judgement are needed for interpretation
Mahmood_1996bA	Clearance, volume of distribution	A new approach incorporating brain weight in the model improves prediction of clearance
Mahmood_1996cA	Clearance, volume of distribution	Three or more species are needed for reliable prediction of clearance, while volume of distribution is predicted equally well using data from two species or more
Mahmood_1998a	Clearance	Mean residence time can be predicted reasonably well for man and can be used for prediction of half-life
Mahmood_1998bA	Clearance, volume of distribution	Caution should be employed when interpreting clearance predictions for renally excreted drugs. Predicted volumes (error − 65.6% to 139.4%) and half-lives (error − 41.8% to 100%) were comparable with observed values in man.
Mahmood_1999	Selection of 1st in human dose	The half-life and bodyweight correlate poorly; body weight is not useful as a predictor
Mahmood_2000a	Bioavailability	All tested approaches predicting human bioavailability from animal data are inaccurate
Mahmood_2000b	Protein binding	Unbound human clearance cannot be predicted any better than total human clearance from animal data
Mahmood_2001	Maximum tolerated dose	Maximum tolerated dose can be predicted with reasonable accuracy using interspecies scaling
Mahmood_2003	Selection of 1st in human dose	Animal PK data from a dose not producing adverse events can be used to estimate a suitable human starting dose
Mahmood_2004	Clearance	More than two species are needed for reliable clearance predictions of protein drugs
Mahmood_2006	Clearance	There is no single method for predicting human clearance from animal data for all classes of drugs
Mahmood_2009	Clearance	Predictions based on at least 3 animal species remain more accurate than one or two-species methods
Mahmood_2012	Clearance, volume of distribution	The human clearance of drugs that are excreted in the bile can be predicted with reasonable accuracy from animal data. The volume of distribution does not appear to be affected by biliary excretion
Mahmood_2013	Concentration–time profiles	Human concentration–time profiles of therapeutic proteins can be predicted reasonably accurate from animal data
Mahmood_2013	Clearance, volume of distribution	Concentration–time profiles are accurately predicted for most time points
Mahmood_2016	Clearance, volume of distribution	Human plasma time–concentration profiles, oral clearance and volume of distribution can be predicted with reasonable accuracy
McGinnity_2007	Clinical dose, maximum concentration & volume of distribution	There is a reasonable to good correlation between projected and clinical human dose, observed and predicted maximum concentration for a given human dose and predicted and observed human volume of distribution
Musther_2014	Oral bioavailability	Bioavailability in animals is not quantitatively predictive of bioavailability in humans
Nagilla_2004	Clearance	Prospective allometric scaling is a suboptimal technique for estimating human clearance data from in vivo preclinical data
Naritomi_2001	Clearance	Animal data improve predictions of human clearance from in vitro liver microsomes
Obach_1997	Volume of distribution, clearance	Methods for accurate prediction of human PKPD based on animal data do not currently exist, but many methods result in adequate predictions
Paine_2011	Clearance	The most accurate predictions of human renal clearance are obtained from a direct correlation with dog renal clearance. Adding data from rats decreases predictability
Pogessi_2004	Volume of distribution, clearance	In most cases, animal-based predictions are within two or threefold of those observed in humans
Rocchetti_2007	Active dose	Therapeutically active concentrations of anticancer drugs can be estimated from preclinical studies
Sanoh_2012	Clearance	PXB chimeric mice can be used for at least semi-quantitative prediction of human clearance and half life
Sanoh_2014	Metabolism	Human metabolites were sufficiently predicted from the animal data for 10 out of 16 compounds; predictions were insufficient for 6 out of 16 compounds
Sawada_1985A	Clearance, volume of distribution	Predictions for human clearance, volume of distribution and half-life from rat data were successful for most drugs, with marked exceptions
Sawada_1985B	Volume of distribution	Prediction of human volume of distribution based on animal plasma unbound fraction was successful for most drugs
Schneider_1999	Clearance	Dog and rat in vivo hepatic drug clearance data appear unrelated with human data
Sietsema_1989	Oral bioavailability	Absolute bioavailability does not correlate well between species
Takahashi_2009	Bioavailability	The bioavailability in cynomolgus monkeys was low compared to that in humans for most drugs tested
Tang_2005	Clearance	A new mathematical model based on unbound fractions can improve prediction of human clearance from animal data
Tang_2006	Clearance	There is no strong evidence that human systemic clearance is more predictable from animal data than human oral clearance
Wajima_2002	Clearance	Multiple linear regression of animal data generally predicts human clearance better than allometric methods
Wajima_2003	Oral clearance	The partial least square method based on animal data generally predicts human oral clearance better than allometric approaches
Walton_2004	Clearance	Average differences in the internal doses between humans and animals were 1.6 for dogs, 3.3 for rabbits, 5.2 for rats and 13.0 for mice
Wang_2010	Clearance	Human clearance can generally be predicted well from animal data with simple allometric scaling
Ward_2004a	Clearance	Generating data in multiple non-human species does not always result in improved prediction
Ward_2004b	Volume of distribution	The monkey provides the most accurate PKPD predictions for humans. The allometric exponent cannot be used as a reliable marker of predictive success
Ward_2005	Clearance	The rat is not as accurate a predictor as the monkey, but still affords reasonable human predictivity
Ward_2005	Oral systemic exposure	Liver-corrected oral exposure was within twofold of human for 30% of compounds for rats, and for 48% for dogs. The monkey was the preclinical species most similar to humans
Ward_2008	Clearance	Reasonable predictive accuracy of pharmacokinetic parameters in humans can be achieved with African green monkeys
Ward_2009	Bioavailability	The African green monkey provides similar predictivity for human oral exposure as other monkeys
Whiteside_2010	Maximum concentration	Rat models for pain predict effective exposure levels in humans. Effective plasma concentrations also correspond.
Wong_2004	Clearance	The chimpanzee serves as a valuable surrogate model for human pharmacokinetics

Several scatterplots of pharmacokinetic parameters for a set of drugs in animals versus humans show low correspondence rates, i.e. they do not show an apparent relationship between animal and human data (e.g. [72, 73]. Of note, these types of plots are specifically sensitive to selection bias; if one is familiar with the literature it is relatively easy to (consciously or subconsciously) select a set of drugs with relatively high or relatively low correspondence. Besides, PK correlational review papers are often based on the same experiments and data; the same data have e.g. been included in [74–76].

### Hypotheses-generating analyses of translational success rates

**Fig. 2:**
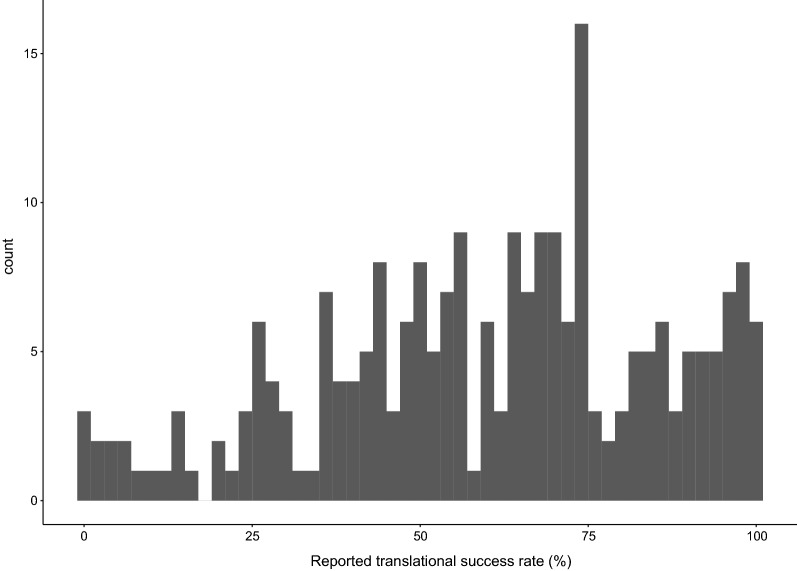
Histogram of the translational success rates (%) in the included studies

The range of published translational success rates is 0% to 100%. A histogram of all published translational success rates is provided in Fig. 2.

**Fig. 3:**
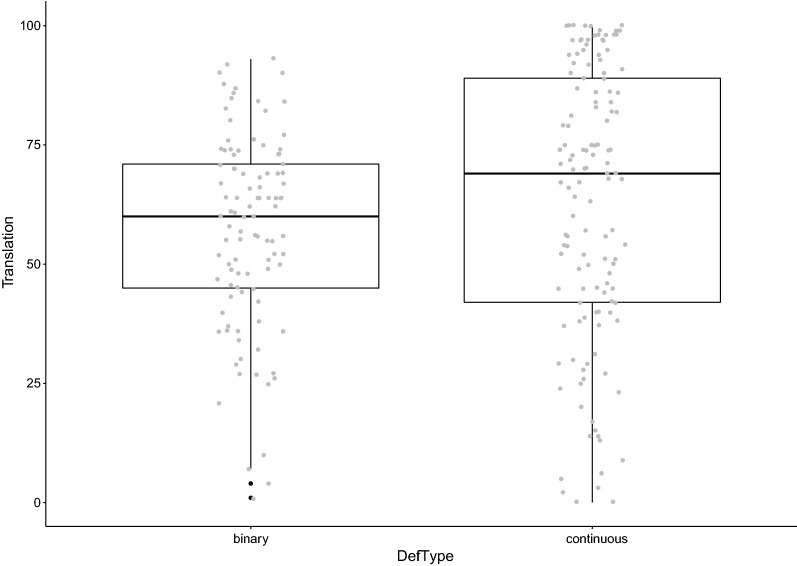
Reported translational success rates (%) by type of definition of translational success (binary vs. continuous diagnostic statistics). *DefType* type of definition

As we included outcomes from different types of analyses, we compared the effect of the two broadly defined definitions of translation; binary (translation successful yes/no) and continuous (amount of correspondence; explained variance) in a boxplot (Fig. 3). For studies using binary definitions of translation, translational success rates ranged from 0 to 93%. Binary definitions comprise the diagnostic statistics fold error (i.e. the percentage of studies/compounds below twofold error), percentage of studies/compounds/adverse events accurately predicted, positive predictive values and negative predictive values. For studies using quantitative definitions of translation, translational success rates ranged from 0 to 100%. Quantitative definitions comprise the diagnostic statistics correlation/regression (r^2^ expressed as a percentage) and meta-analyses (percentage overlap of 95% confidence intervals of the summary measure). The outcomes of analyses of translational success could be affected by the choice of definition, but the range is large either way.

**Fig. 4:**
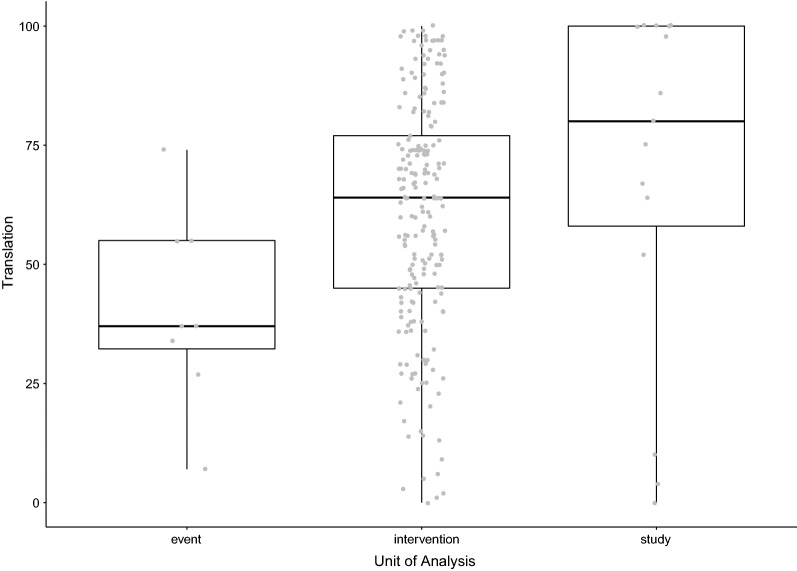
Reported translational success rates (%) by analysis unit

As we included reviews using different units of analyses, we compared the effect of the unit: event (e.g. a specific adverse event), intervention (mostly drugs) or study (or publication) on translational success rates in a boxplot (Fig. 4). For the 8 studies analysing events, the translational success rate ranged from 7 to 74%. For the other two units of analysis, ranges comprised the full spectrum of 0–100% translational success.

**Fig. 5:**
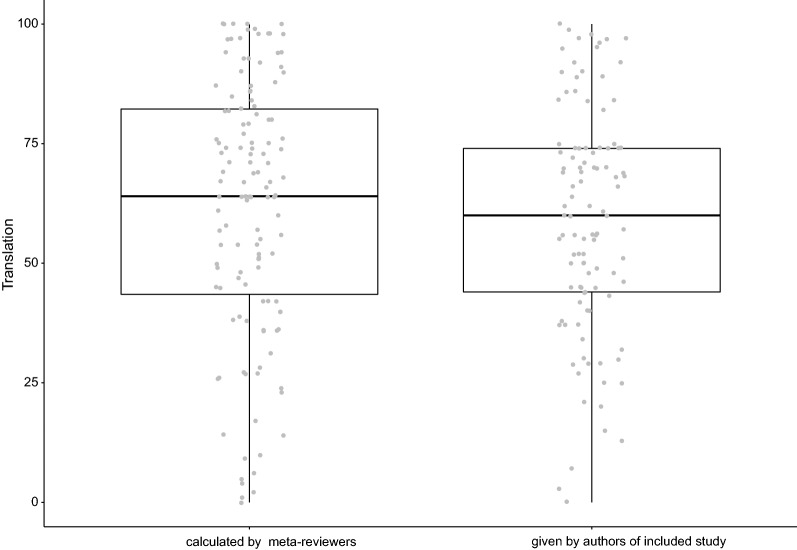
Reported translational success rates (%) by calculators: the original authors vs. the meta-reviewers

We copied the translational success rates from the authors where possible, but also included papers for which the summary measure of interest was not directly given (e.g. manually calculating a correlation or a percentage overlap in 95% CI, refer to the methods for further information). We visually compared the percentages calculated by us with those calculated by the authors of the included papers in a boxplot (Fig. 5). Both categories comprised the full range of 0–100% translational success.

**Fig. 6:**
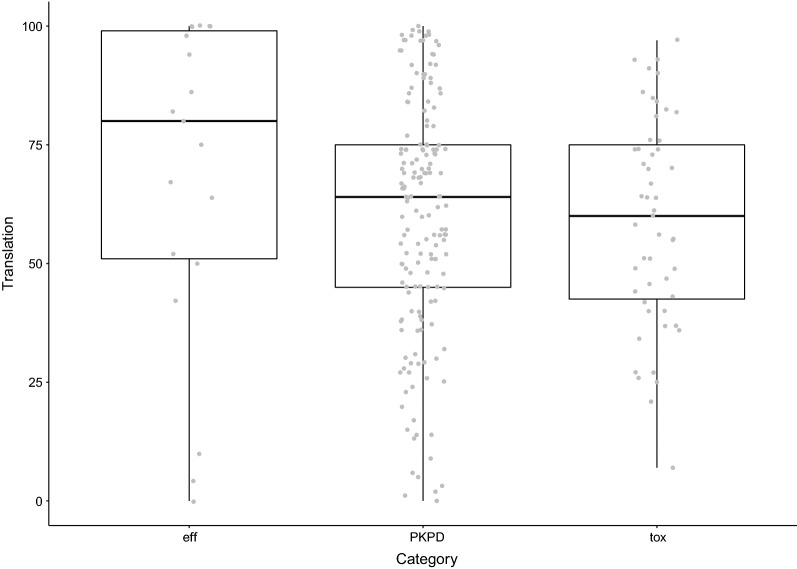
Reported translational success rates (%) by broadly defined research category. *Eff* efficacy, *PKPD* pharmacokinetics or pharmacodynamics, *tox* toxicology

We then grouped the included studies into broad research categories: toxicology, PKPD and efficacy. Translational success rates by category are shown in Fig. 6. No clear differences are observed between these categories. Differences may still be present between more precisely defined medical fields (e.g. cardiovascular disease, neuroscience, inflammation, oncology), but in-depth analysis of differences in translational success rates between these fields is not possible based on the available data, as most fields have been analysed only once or twice.

**Fig. 7:**
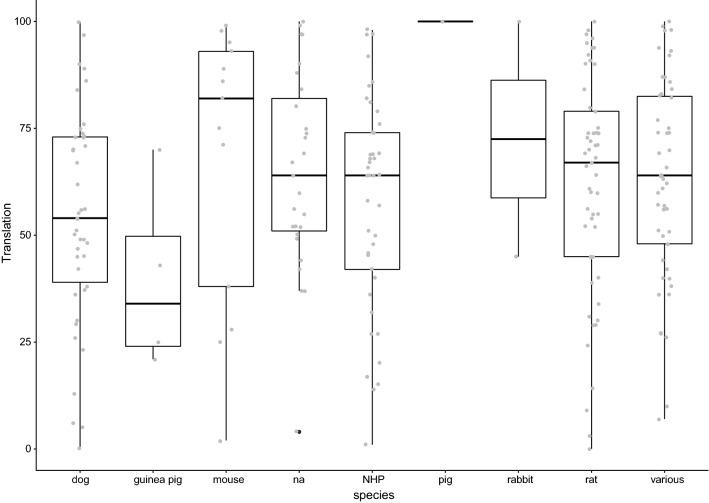
Reported translational success rates (%) by species. *NA* information on species not available

We next grouped the included studies by species. Translational success rates by species are shown in Fig. 7. Several references did not specify the species used, several others only presented data from several species pooled. Only few studies were available on guinea pigs, only one on pigs. No clear differences are observed between species.

**Fig. 8:**
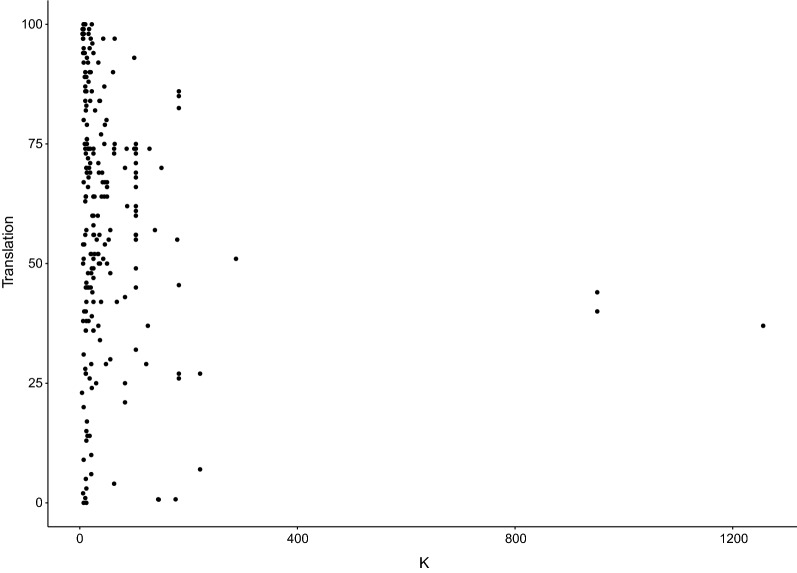
Reported translational success rates (%) by study size. K = the number of included compounds/interventions, studies/experiments, or symptoms/events

Our next analysis shows the reported translational success rates by study size (i.e. number of included compounds/interventions, studies/experiments, or symptoms/events, all referred to as K, Fig. 8). The studies with n > 200 are all toxicology studies using a binary definition of translation and have been described above.

**Fig. 9:**
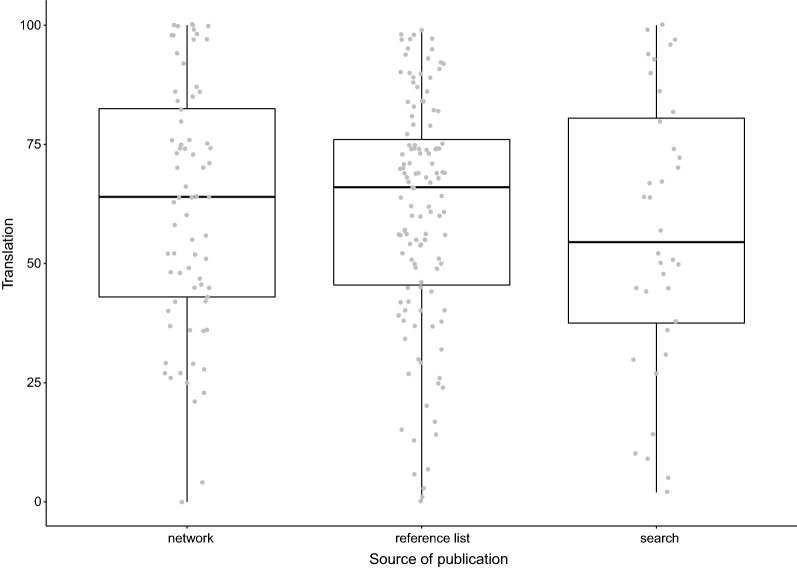
Reported translational success rates (%) by paper source

To test the potential effects of the various search strategies used, we compared the translational success rates between studies retrieved via our network, reference lists and database searches. Translational success rates by source are shown in Fig. 9. No clear differences are observed between these sources; all ranges comprise translational success rates of 2–99%.

**Fig. 10:**
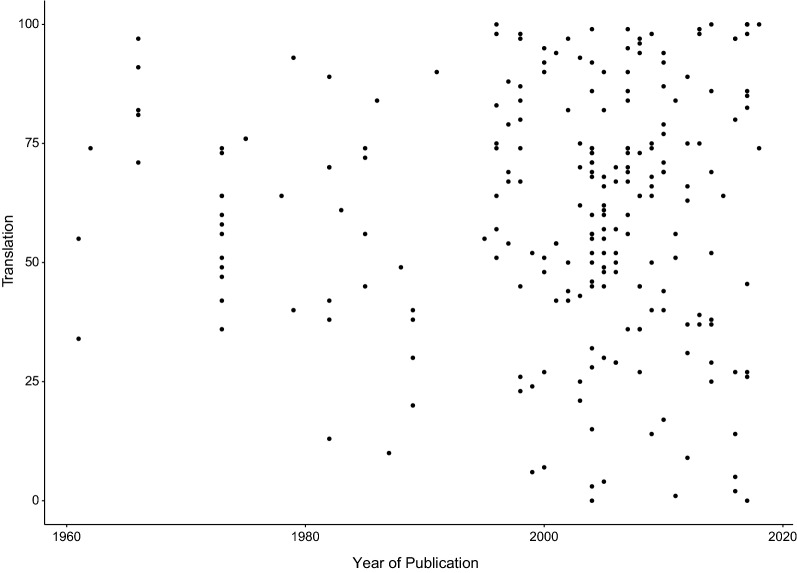
Reported translational success rates (%) by publication date

Our last analysis shows the reported translational success rates by publication date (Fig. 10). We observe an increase of both the numbers of studies and the observed range of translational success over time.

### Risk of bias and reporting quality

**Fig. 11:**
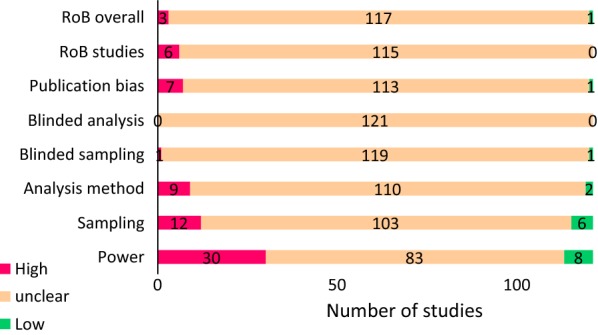
Summary of risk of bias of the included studies. Numbers are absolute values

Our analysis of the reporting quality of the included references and the risk of bias is summarised in Fig. 11. Many details of the review designs were poorly reported, resulting in an overall unclear risk of bias for our scoping review.

Reporting of the selected PRISMA-items was also poor; none of the references described the posting of a protocol, the number of people screening the papers, the number of people extracting the data, or prespecifying the analyses. Specific eligibility criteria were described in 31 out of the 121 references (26%), limitations by 37 out of the 121 references (31%).

Out of the 121 references, 27 contained specific information on the funding. Risk of funding bias could work in two directions; studies funded by animal rights organizations are expected to find lower than average translational success rates while those with funding from pharmaceutical companies and governmental organizations may be more inclined to overestimate translational success. If we include the affiliations of the authors in our risk of bias assessment for the funding, 81 out of the 121 references had a high risk of funding bias.

## Conclusion

### General considerations

This systematic scoping review of reviews provides an overview of research efforts on translational success rates. It shows that the amount of available evidence and the overall quality are limited, and that there is high variability between study types. The published translational success rates range from 0 to 100%. The wide range of translational success rates observed in our study, and the lack of a clear relationship with any of the analysed factors, might indicate that translational success is unpredictable; i.e. it might be unclear upfront if the results of primary studies will contribute to translational knowledge. However, the risk of bias of the included studies was high, and much of the included evidence is older (note that this is a review of reviews, the most recent included reviews will be based on older data), while newer models have become available. Therefore, the cumulative evidence of current papers on this topic is insufficient and further “umbrella”-studies of translational success rates are still warranted.

We included studies on animal-to-human translation. We originally defined successful translation as replication in a randomized trial in humans of statistically significant positive, negative or neutral results for the primary study outcome in animal experiments. However, we did not define “replication”. When writing the protocol, we intended to include studies based on systematic reviews [59], animal ethics requests [58] and highly cited animal publications [57]. The set of included studies however also comprises many correlational and modelling PK studies, in line with our adapted definition of translation: “the quantitative degree of correspondence between the results from a trial in humans with results in animal experiments”.

We do not see a difference in predictivity between toxicology, PKPD and efficacy studies. Before we ran the analyses, we expected the toxicology studies to be more predictive than the efficacy studies, first, because toxicology may reflect more conserved physiological mechanisms, second, as toxicology studies are generally performed in multiple species, and third, as toxicology studies are generally performed according to Good Laboratory Practice standards, resulting in higher internal validity of the results.

### Search

Scoping searches taught us that a full comprehensive search strategy would result in large numbers of retrieved references, with limited sensitivity. As the resulting amount of work was not manageable within a reasonable time frame, we opted to perform a scoping review instead of a full systematic review, with an in-depth analysis of a subset of the literature.

Our search was thus based on thesaurus (i.e. indexed) and title words only, resulting in missing those papers only describing translation or predictivity in the abstract or the text body while not being indexed for them. We supplemented our search with alternative strategies, i.e. screening the reference lists, contacting first and last authors, and contacting our network, to compensate. We retrieved more references via these alternative strategies (i.e. 60 + 35 = 95, Fig. 1) than via our searches (i.e. 26, Fig. 1), underlining the need for improved indexing of this type of studies.

Snowballing via reference lists is not an optimal method in this field, first because referencing practices are suboptimal (compare e.g. the data and figures from [5, 72, 73, 77]. Second, many studies focussing on alternatives to animal studies also contain information that quantitatively compares animal and human data (e.g. [78] and these relevant studies are difficult to identify from their titles.

A limitation of our search is that we did not include a term for modelling and scaling studies, as we did not have this type of study in mind at the time of designing our protocol. These studies may not specify translation or prediction in their title, e.g. [79, 80]. While using these terms will increase the number of irrelevant hits, to be complete, we do recommend adding the terms “modelling”, “scaling”, “correlation” and their synonyms to retrieve these papers in future searches for translational studies.

A further limitation is that we performed our search halfway October 2017, which is rather inherent to the systematic approach. Systematic reviews of clinical studies take on average 67.3 weeks from registered start to publication [81]. The alternative supplementary strategies increase the review duration, as screening of reference lists and contacting authors of the included studies could only be finalised after full-text screening had been completed and discrepancies between reviewers had been resolved.

We are aware that not all available evidence has been included. To prevent eternal snowballing and to finish this review in a timely manner, we decided to stop retrieving further references from the second-line reference lists onwards. During data-extraction, our occasional checks of reference lists of the later included papers showed that most studies had already been included, indicating that, for a scoping review, our data-set can be considered as almost complete.

A full systematic review following the methodology described in this scoping review would probably result in a larger data set. However, we cannot envision our alternative search strategies to be biased towards a certain outcome. Contacting the authors of the papers retrieved by the search is relatively objective and reproducible. The authors’ network should not induce substantial bias either, as the opinions on translational success rates between the authors vary. The main outcome of this study is the observed range of translational success rates. As this comprises all possible values (0–100%) it could not change because of more complete sampling strategies.

### Data quality and risk of bias

Some of the general issues with analysing translational failure and success rates have been described before [82]. Besides, our analyses are affected by several factors. Factors generally affecting the quality of scoping reviews comprise publication bias, unblinded data selection, unblinded extraction, unblinded analysis and statistical power. Publication bias is the relative underreporting of studies not showing a significant effect in scientific literature. For the observed range of reported translational success rates, from 0 to 100%, we do not consider publication bias a specific concern. We strove to limit bias in the inclusion of data by having two reviewers select papers independently. Of note, the observed range of translational success was not drastically affected by publication date or manner of publication retrieval. Data extraction and analysis were not performed in a blinded manner, but the extractor (CHCL) had no a priori expectations on the results. As data were not quantitatively analysed, statistical power is not relevant.

Besides, several factors specifically affect the quality of the data included in this work. The first is the problem with dependency of the data; several authors and research groups publish multiple papers on translational success rates, often based on (partially) overlapping data sets. For example, Schein present an analysis of 25 anticancer drug toxicities in several papers [83–86], each paper combining the analysis with other information. For our quantitative analyses, we aimed for incorporating each dataset only once, but if datasets only overlapped partially, they were both included.

The second is that we included several measures for translational success, based on different definitions, using different diagnostic statistics. We classified the different measures into two broad categories, based on the underlying definition of translational success, which could be binary (yes/no) or continuous (% concordance), and did not observe large differences in observed translational success rates between these categories. Translational success rates were also not affected by unit of measurement (event, intervention or study) used in the original review, or by who did the calculations (us or the authors of the review). However, the percentage overlap in CIs, which we used to describe translational success for meta-analyses, is disputable for two reasons. First, the overlap in CIs could be fair even if the estimates are quite far apart if both estimates are unprecise. We consider the CIs of the included studies not to be that large. Second, many scientists argue that the size of the effect is irrelevant as long as the direction of the effect is the same. As described in our methods, for the included meta-analyses, only one set of CIs, from rats and humans, did not overlap [65]. The direction of the effect here was opposite, and we included it in our analyses as 0% concordance. We preferred including the percentage overlap over excluding the meta-analyses from our review, and excluding this paper would not have affected the overall observed range of translational success.

A third factor is that dosing and incidence of events are often disregarded [82]. Concerning dosing, differences in metabolism, weight, distribution volume etc. result in different dosing, and oversimplified approaches for dose prediction are common [87]. Concerning incidence, known human carcinogens may be tested more extensively in animals than compounds without known human toxicity, eventually showing positive results in at least one preclinical test. Besides these factors, publication bias (i.e. the relative underreporting of primary studies with negative and neutral results) can obscure translational failure rates [13].

Our analysis of risk of bias in the included references shows an overall unclear risk of bias, with a high risk of funding bias for 81 out of the 122 included references. Besides, there was a high risk for underpowered studies in 30 out of 121 included references. Our analysis of the reporting quality of the included studies showed that most reviews did not comply with the PRISMA guidelines, but this is not unexpected, as most of the included references did not claim to be systematic reviews.

Many of the included reviews had drugs as the experimental unit. Most of these did not describe their selection of the drugs, explicit inclusion and exclusion criteria were scarce. One that did transparently describe their selection procedure excluded studies with novel targets, where predictivity is most needed [88]. This same study shows that analysing a subset excluding outliers can increase the predictivity of the animal studies.

To conclude, the data presented in this paper have severe limitations. They should be considered inconclusive and used for hypothesis-generation only. Besides, reliably determining actual translational success rates is unmanageable as long as the current status of reporting of preclinical research leaves room for improvement [89], and non-reproducibility is such a critical issue in both animal [1] and human [90] studies.

### Implications

While the quantity and quality of the available data is limited and further studies are still needed, this review provides an at least relatively complete overview of published evidence on translational success rates. These actual numbers for predictiveness are theoretically more informative than qualitative, subjectively determined, mechanistic similarities between animal models and human pathology. Therefore, for animal studies aimed at translation to the human situation, where possible, probabilistic evidence for predictivity should be considered besides or even instead of mechanistic evidence.

Of note, animal studies may contribute to successful translation in other manners than direct prediction of the human response; they can for example be informative in hypothesis-generation for mechanisms underlying disease. We emphasise that this review does not provide any information on the usefulness of animals in fields of animal use that do not directly target predictivity for humans.

To ensure validity of the gathered animal and human data, it is essential that the execution of the studies is of high quality, and that the reporting is complete. Complete reports of high-quality studies are needed to determine actual translational success rates, and to identify factors involved in translational success. Knowing the factors involved in translational success will benefit both animals and humans.

## Data Availability

All included data are available in the public domain and all references are included in our reference list. Extracted data and calculations will be made available to individual scientists upon reasonable request.

## Abbreviations

AN: animal negative
AP: animal positive
CI: confidence interval
FN: false negative
FP: false positive
HN: human negative
HP: human positive
LR: likelihood ratio
MeSH: medical subject heading
NPV: negative predictive value
PK: pharmacokinetic
PKPD: pharmacokinetic–pharmacodynamic
PPV: positive predictive value
PRISMA: preferred reporting items for systematic reviews and meta-analyses
SR: systematic review
TN: true negative
TP: true positive
